# Trustworthy causal biomarker discovery: a multiomics brain imaging genetics-based approach

**DOI:** 10.1093/bioinformatics/btaf257

**Published:** 2025-07-15

**Authors:** Jin Zhang, Yan Yang, Muheng Shang, Lei Guo, Daoqiang Zhang, Lei Du, Alzheimer’s Disease Neuroimaging Initiative, Jin Zhang, Yan Yang, Muheng Shang, Lei Guo, Daoqiang Zhang, Lei Du

**Affiliations:** Department of Intelligent Science and Technology, Northwestern Polytechnical University School of Automation, Shaanxi 710072, China; Department of Intelligent Science and Technology, Northwestern Polytechnical University School of Automation, Shaanxi 710072, China; Department of Intelligent Science and Technology, Northwestern Polytechnical University School of Automation, Shaanxi 710072, China; Department of Intelligent Science and Technology, Northwestern Polytechnical University School of Automation, Shaanxi 710072, China; School of Artificial Intelligence, Nanjing University of Aeronautics and Astronautics, Nanjing 210000, China; Department of Intelligent Science and Technology, Northwestern Polytechnical University School of Automation, Shaanxi 710072, China

## Abstract

**Motivation:**

Discovering genetic variations underpinning brain disorders is important to understand their pathogenesis. Indirect associations or spurious causal relationships pose a threat to the reliability of biomarker discovery for brain disorders, potentially misleading or incurring bias in subsequent decision-making. Unfortunately, the stringent selection of reliable biomarker candidates for brain disorders remains a predominantly unexplored challenge.

**Results:**

In this article, to fill this gap, we propose a fresh and powerful scheme, referred to as the Causality-aware Genotype intermediate Phenotype Correlation Approach (Ca*-*GPCA). Specifically, we design a bidirectional association learning framework, integrated with a parallel causal variable decorrelation module and sparse variable regularizer module, to identify trustworthy causal biomarkers. A disease diagnosis module is further incorporated to ensure accurate diagnosis and identification of causal effects for pathogenesis. Additionally, considering the large computational burden incurred by high-dimensional genotype–phenotype covariances, we develop a fast and efficient strategy to reduce the runtime and prompt practical availability and applicability. Extensive experimental results on four simulation data and real neuroimaging genetic data clearly show that Ca*-*GPCA outperforms state-of-the-art methods with excellent built-in interpretability. This can provide novel and reliable insights into the underlying pathogenic mechanisms of brain disorders.

**Availability and implementation:**

The software is publicly available at https://github.com/ZJ-Techie/Ca-GPCA.

## 1 Introduction

Alzheimer’s disease (AD) is a complex neurological disorder that imposes substantial economic and emotional burdens on patients and their families (Shen and Thompson 2019, Sims *et al.* 2020). Emerging evidence underscores the pivotal role of reliable biomarkers in AD diagnosis, prevention, and treatment (Lu *et al.* 2020a,b, Wang *et al.* 2023). Regrettably, the practical translation of computational findings into clinical applications remains a formidable challenge in machine learning, largely due to the lack of reliable causal biomarker candidates. Therefore, identifying trustworthy causal biomarkers, i.e. genetic factors or endophenotypes could handle this and further contribute to a deeper systematic understanding of AD (McCarthy *et al.* 2008, Manolio *et al.* 2009, Eichler *et al.* 2010, Simons *et al.* 2018, Zhang *et al.* 2024b).

**Figure 1.**
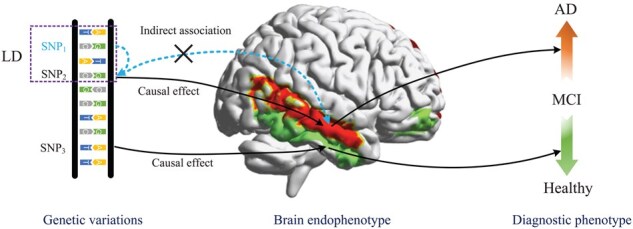
A schematic illustration of our work. ${\text{SNP}}_{1}$ is erroneously identified as a risk factor for disease-related phenotype due to the linkage disequilibrium (LD) relationship to ${\text{SNP}}_{2}$, and the influence of this LD could be inadvertently inflated. This spurious causal effect can introduce biases in subsequent analysis. Therefore, we propose a causality-aware bidirectional association learning framework to remove indirect associations and exploit causal contributions for brain disorders.

Brain imaging genetics has offered a powerful avenue for gaining profound insights into the genetic underpinnings of brain intermediate phenotypes, i.e. endophenotypes (Shen and Thompson 2019, Chung *et al.* 2023). Over the past decade, a variety of approaches, including univariate, multivariate, and bi-multivariate methods, have been proposed to unravel the intricate interplay between genetic variations and endophenotypes (Wang *et al.* 2012, Lin *et al.* 2014, Wei *et al.* 2014, Zhang *et al.* 2022b, 2024a). However, these methodologies fall short of confirming the true causal effects for diagnostic outcomes, hampering or even misleading the biological interpretation (Zille *et al.* 2017, Kuang *et al.* 2018, Lisowska *et al.* 2019, Du *et al.* 2020a,b). For example, the presence of linkage disequilibrium (LD) poses a challenge to discerning causal risk variations, largely due to the tight correlation, or dependence, among genetic variations during the cell meiosis stage, which finally impedes the precise identification of causative factors (Sesia *et al.* 2020, LaPierre *et al.* 2021, de Los Campos *et al.* 2023). Figure 1 shows a schematic example. The causal genetic variation (single-nucleotide polymorphism [SNP], in this article) refers to the true association signal at a given genetic locus. If an irrelevant SNP is erroneously identified as triggering the disease-related phenotype due to its high association with a true causal SNP, the influence of this irrelevant SNP and the heritability of the disease could be inadvertently inflated (Lonjou *et al.* 2003). These introduce substantial biases for brain disorders, especially AD (Shen and Thompson 2019, Chung *et al.* 2023). Hence, enhancing the ability to identify significant and reliable causal genetic variations, while also elucidating causal relationships between genotypes and endophenotype, becomes an urgent need for AD (Du *et al.* 2017, Guo and Wu 2019, Sheng *et al.* 2019, Zhao *et al.* 2020, Du *et al.* 2021).

There are two significant difficulties in the identification of causal effects. First, which has been shown above, neighboring SNPs are often highly correlated, due to the LD of shared ancestry (Sesia *et al.* 2020). This results in an indirect association between an SNP and disease outcome in imaging genetic studies (Udler *et al.* 2010, Wainberg *et al.* 2019, Novo *et al.* 2022), and is likely to lead to spurious findings, thus potentially biasing subsequent decision-making of healthcare (Casale *et al.* 2015, Hibar *et al.* 2017, Kuang *et al.* 2017). Second, addressing these spurious genotype–phenotype (GP) causal effects poses computational challenges. Although covariate balancing methods, such as propensity score matching or variable balancing, could be a solution to estimate causal effects (Rosenbaum and Rubin 1983, Hainmueller 2012, Li and Fu 2017, Fong *et al.* 2018), ensuring perfect variable balancing is still challenging when dealing with high-dimensional features with limited samples. Structural causal models (SCMs) provide a robust framework for identifying causal variables via conditional independence tests but encounter substantial challenges in high-dimensional genomic applications. Moreover, eliminating unstable variables in such scenarios is also difficult, potentially leading to underperformance and imposing computational burden (Solovieff *et al.* 2013, Casale *et al.* 2015).

To address the abovementioned problems, we propose a simple yet versatile framework, Ca-GPCA. In particular, we incorporate parallel causal variable decorrelation and sparsity regularized modules into a bidirectional association learning framework. This can enhance interpretability and reliability while preserving predictability. First, the bidirectional association learning framework makes it easier and more reasonable to incorporate genetic variations and endophenotypes simultaneously. Second, we meticulously devise the parallel causal variable decorrelation to eliminate indirect associations to boost GP correlations, as well as effectively mitigate computational challenges. Third, to ensure causal biomarker identification and facilitate interpretation, we introduce an interpretable regularized module to select important features and remove unstable features, which help recover causality between genetic variations and phenotypes. Fourth, we design a disease diagnosis module to ensure the identified biomarkers’ relevance to the disease of interest. In addition, since computing high-dimensional GP correlations is very time-intensive, we develop a fast optimization algorithm in the spirit of divide-and-conquer. In conclusion, experimental results on simulated and real neuroimaging genetic data reveal that our method attains superior correlation coefficients and classification accuracy compared to state-of-the-art methods, especially in the identification of causal effect detection (Witten and Tibshirani 2009, Hu *et al.* 2017, Rodosthenous *et al.* 2020). This outcome holds the promise for advancing insights into AD and facilitating targeted therapeutic interventions (Kulminski *et al.* 2020). In summary, primary contributions can be concluded as follows:

- We propose a simple but efficient scheme to identify trustworthy causal biomarkers and facilitate AD prediction or prevention, shedding light on brain imaging genetics.
- We organically combine parallel causal decorrelation, sparsity variable selection, and divide-and-conquer modules iteratively to alleviate the underperformance caused by indirect associations, as well as reduce time complexity, memory usage, and computational difficulty.
- We extend the proposed approach to a more challenging setting, i.e. chromosome-wide setting. This extension enhances the practical applicability of our method, enabling its utilization with large-scale brain imaging genetics data.
- Comprehensive experimental results, encompassing four simulation datasets and real datasets, highlight the consistent and significant performance of our approach compared to state-of-the-art methods.

## 2 Related works

### 2.1 Brain imaging genetics

Brain imaging genetics is a rapidly developing yet challenging field that can comprehensively investigate the genetic architectures of brain endophenotypes (Wang *et al.* 2012, Lin *et al.* 2014, Wei *et al.* 2014, Shen and Thompson 2019). In the past decade, univariate, multivariate, and bi-multivariate techniques have been harnessed to unravel the interplay between genetic variations and intermediate phenotypes of interest (Shriner *et al.* 2007, McCarthy *et al.* 2008, Wang *et al.* 2012, Lin *et al.* 2014, Wei *et al.* 2014). Pairwise univariate analysis rapidly provided critical associations between SNPs and endophenotypes (McCarthy *et al.* 2008). Regression-based methods investigated the joint effect of multiple SNPs on a few candidate endophenotypes (Wang *et al.* 2012). Bi-multivariate methods investigated multiple SNPs and multiple endophenotypes simultaneously (Shriner *et al.* 2007, Lin *et al.* 2014, Wei *et al.* 2014). These methods could not discriminate the causal SNPs, and thus substantial SNPs were substantiated as spurious factors finally (Eichler *et al.* 2010, Simons *et al.* 2018, Cui and Athey 2022).

### 2.2 Trustworthy variable selection

Variable selection, concretely biomarker discovery, is a notable domain in machine learning. Causal inference served as a potent statistical modeling tool for selecting explanatory variables (Rosenbaum and Rubin 1983, Hainmueller 2012, Li and Fu 2017, Fong *et al.* 2018), i.e. Rubin *et al.* advocated for achieving equilibrium through propensity score matching or sample reweighting, while Hainmueller *et al.* introduced entropy balancing as a means to directly adjust sample weights based on specified sample moments (Rosenbaum and Rubin 1983, Hainmueller 2012, Li and Fu 2017). Nevertheless, these methods are constrained by a fundamental limitation. When confronted with high-dimensional features and limited sample sizes, variable selection can manifest substantial variability, thereby compromising confidence in their relevance to diagnostic outcomes (Yang *et al.* 2023). The challenge arising from high dimensionality poses obstacles to both sparsity and reliability, consequently influencing the interpretability of predictive models. Therefore, we aim to introduce an efficient scheme for identifying causal and trustworthy biomarkers while preserving predictivity.

## 3 Materials and methods

### 3.1 Ca*-*GPCA

As mentioned above, the rigorous selection of trustworthy causal biomarkers is essential yet largely unexplored in imaging genetics. We thus propose a simple and effective framework to identify causal genetic markers for brain endophenotypes, and further facilitate AD prediction. For ease of representation, genetic variations are denoted as $X\in R^{n\times p}$, the *f*th brain endophenotypes are denoted as $Y_{f}\in R^{n\times q_{f}}$, z is diagnostic status, where *n* is number of subjects, p is number of SNPs, $q_{f}$ is number of *f*th endophenotype features, i.e. imaging, proteomic, or cognition phenotypes. Then Ca*-*GPCA is formulated as follows:


(1) $$\begin{array}{c} \underset{u_{f},Q,v_{f}}{\min}\sum_{f=1}^{F}-u_{f}^{⊤}X^{⊤}\mathrm{diag}(Q)Y_{f}v_{f}+\psi \left(v_{f};Y_{f},z\right)\\ +\eta \sum_{k=1}^{K}\sum_{i,j\in \Pi_{i}}\left(\mathrm{causal}\;{\left(X_{i},X_{j};Q\right)}^{2}\right).\\ \;s.t.\;{\left\|Xu_{f}\right\|}_{2}^{2}=1,{\left\|Y_{f}v_{f}\right\|}_{2}^{2}=1,\Omega \left(u_{f}\right)\leq c_{1},\Omega \left(v_{f}\right)\leq c_{2},\Omega (Q)\leq c_{3}. \end{array}$$

$U=[u_{1},\ldots ,u_{F}]\in R^{p\times F}$ is canonical weight carrying the effects of SNPs, $V=[v_{1},\ldots ,v_{F}]\in R^{q\times F}$ is that carrying weights of endophenotypes, and $Q\in R^{n\times 1}$ is a sample weight vector learned from causal decorrelation module to remove SNPs with indirect associations. Ω(U) and Ω(V) are penalty terms for biomarkers detection.

**Figure 2.**
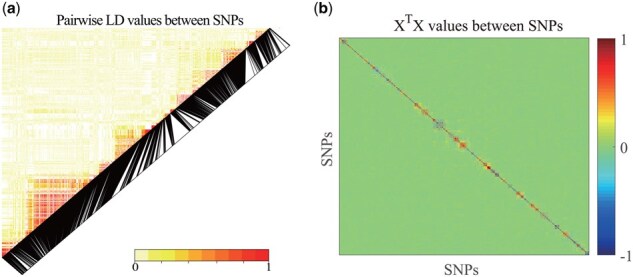
Illustration of the pairwise correlation coefficients and LD values of SNPs from ADNI database. (a) Pairwise LD values. (b) $X^{⊤}X$ values among SNPs.

In our model, the first term is a bidirectional association learning module to learn GP correlation. $\psi \left(v_{f};Y_{f},z\right)$ is disease diagnosis module based on a metric function (linear regression loss, $\psi_{\mathrm{lin}}\left(v_{f};Y_{f},z\right)=\frac{1}{2}\sum_{i=1}^{n}{\left(z_{i}-\langle Y_{f_{i}},v_{f}\rangle \right)}^{2}$ logistic regression loss, $\psi_{\;\text{log}\;}\left(v_{f};Y_{f},z\right)=\frac{1}{2}\sum_{i=1}^{n}\left(\text{log}\;\left(1+\text{exp}\;\langle Y_{f_{i}},v_{f}\rangle \right)-z_{i}\langle Y_{f_{i}},v_{f}\rangle \right)$), to ensure the detected biomarkers to be disease-related. The synergy between these two components enhances both disease prediction accuracy and biomarker interpretability. Therefore, assigning equal optimization weight ensures that neither task is deprioritized, leading to a more robust and interpretable model. The causal decorrelation module $\left(\mathrm{causal}{\left(X_{i},X_{j};Q\right)}^{2}\right)$ prompts the independence of $X_{i}$ and $X_{j}$ after being reweighted, which focuses on removing correlations among features, η is a hyperparameter to balance its contributions. In practice, feature decorrelation is difficult given the huge numbers of SNPs in the human genome. However, opportunities and challenges coexist. As shown in Fig. 2, SNPs naturally exhibit a block structure rather than a random one. That is, nearly all of the high correlation or collinearity exists within LD blocks. Thus, we divide all SNPs into *K* groups by LD structures $\prod ={\left\{\pi_{k}\right\}}_{k=1}^{K}$, such that ${\left\{X_{i}\right\}}_{i=1}^{p_{k}}\in \pi_{k}$ and $p_{k}$ is the number of SNPs in group $\pi_{k}$. Now we merely need to remove the collinearity within each LD block, which can be done in parallel. We define our parallel causal decorrelation module as follows:


(2) $$L_{\text{causal}}=\sum_{k=1}^{K}\sum_{i,j\in \Pi_{k}}{\left\|E\left(X_{i}^{T}\Sigma_{Q}X_{j}\right)-E\left(X_{i}^{T}Q\right)E\left(X_{j}^{T}Q\right)\right\|}_{2}^{2},$$

where $\Sigma_{Q}=\mathrm{diag}(Q_{1},\ldots Q_{n})$ is the corresponding diagonal matrix. Hence, our approach could effectively mitigate computational challenges by eliminating the collinearity among SNPs within each LD block, and the causal SNPs will be well preserved. Of note, SNPs within the same LD block are grouped together, where correlation coefficient more than 0.2.

Moreover, the limited sample size complicates the removal of spurious causal effects, hindering the estimation of optimal weights (Zhao and Yu 2006). Thus, we propose a hybrid sparsity penalty that enhances both group and individual feature selection, ensuring stability and interpretability in finite-sample settings:


(3) $$\Omega (U)=\lambda_{u_{l}}\|U{\|}_{\mathrm{FG}L_{2,1}}+\lambda_{u_{2}}\|U{\|}_{2,1}+\lambda_{u_{3}}\|U{\|}_{1,1},$$

${\text{FGL}}_{2,1}$ -norm ($\left||U\right|{|}_{{\text{FGL}}_{2,1}}=\sum_{i=1}^{p-1}\sqrt{\left||u^{i}\right|{|}_{2}^{2}+\left||u^{i+1}\right|{|}_{2}^{2}}.$) here emphasizes the group-wise effect of SNPs, because there exist the inherent group structures among numerous SNPs in the genome. $\ell_{2,1}$-norm is introduced to select consistent features across GP correlation simultaneously. We also use $\ell_{1}$-norm to identify SNPs relevant to specific phenotypes. $\lambda_{u_{1}}$, $\lambda_{u_{2}}$, and $\lambda_{u_{3}}$ are hyper-parameters. We use $\ell_{2}$-norm with respect to Q for a stable solution, and $\sum_{n=1}^{n}Q_{i}=n$ avoids all the sample weights to be zero. In addition, we use $\ell_{1}$-norm to select essential and interpretable phenotypes. Therefore, this joint feature selection holds a diverse and interpretable causal biomarker identification.

### 3.2 Extension to chromosome-wide analysis

Because there are a huge number of SNPs in the human genome, applying Ca-GPCA to genome-wide analysis could be a very challenging task, primarily due to the matrix inversion operation, i.e. $X^{⊤}X$. To mitigate this issue, we design a fast and effective strategy in the spirit of divide-and-conquer, i.e. calculating GP associations separately for each chromosome and then combining the results. Concretely, as shown in Fig. 2, the primary information of $X^{⊤}X$ are well preserved in diagonal LD block since the off-block-diagonal elements approach zero. On this account, we partition the high-dimensional SNPs into *K* non-overlapping subsets, i.e. $U={⊕}_{k=1}^{K}U^{k}$. Of note, the value of *K* is adaptable according to LD structures. Specifically, we partition the large genotype matrices into smaller ones aligned with LD block dimensions, leveraging SNPs’ inherent block structure to maintain model performance. Consequently, the Ca-GPCA objective is redefined as follows:


(4) $$\begin{array}{c} \underset{u_{f},Q,v_{f}}{\min}-\sum_{f=1}^{F}{\left(u_{f1}\;⊕\;u_{f2}\;⊕\;\cdots \;⊕\;u_{fK}\right)}^{⊤}X^{⊤}\;\mathrm{diag}(Q)Y_{f}v_{f}+\psi \left(v_{f};Y_{f},z\right)\\ +\eta \sum_{k=1}^{K}\sum_{i,j\in \Pi_{k}}{\left\|E\left(X_{i}^{T}\Sigma_{Q}X_{j}\right)-E\left(X_{i}^{T}Q\right)E\left(X_{j}^{T}Q\right)\right\|}_{2}^{2}\\ +\Omega \left(u_{f}\right)+\Omega \left(v_{f}\right)+\Omega (Q)\;\;s.t.\;{\left\|Xu_{f}\right\|}_{2}^{2}=1,{\left\|Y_{f}v_{f}\right\|}_{2}^{2}=1 \end{array}$$

Here, ⊕ represents the matrix concatenation operation. We can deduce the closed-form solution of U based on divide-and-conquer. Specifically, $U={⊕}_{k=1}^{K}U^{k}$ with each $U^{k}$ as:


(5) $$U^{k}={\left(\lambda_{u_{1}}D_{u_{1}\text{gk}}+\lambda_{u_{2}}D_{u_{2}\text{gk}}+\lambda_{u_{3}}D_{u_{3}\text{gk}}+\gamma_{1}^{\prime }{\left(X^{⊤}X\right)}_{\text{gk}}\right)}^{-1}X_{\text{gk}}^{⊤}Y,$$

where $D_{u}{}_{g_{k}}$ represents the *k*th diagonal block of $D_{u}$. Consequently, the computational burden is significantly reduced without compromising model performance, thanks to the natural block structure of SNPs. Our approach proves practical for chromosome-wide or genome-wide analysis, supported by the following theoretical analysis.

Algorithm 1 The Ca-GPCA algorithm

**Require:**

The SNP data $X\in R^{n\times p}$, brain endophenotypes $Y_{f}\in R^{n\times q_{f}}$, diagnostic phenotype z. The tradeoff parameters η, γ, $\lambda_{u}$,$\lambda_{Q}$, $\lambda_{v}$, the learning rate α.

**Ensure:**

Canonical weights U, Q, and V.

**1:** Initialize $U\in R^{p\times f}$, $Q\in R^{n}$, $V\in R^{q\times f}$.

**2: while** not convergence **do**

**3:** Solve U according to Eq. (5) by fixing Q and V.

**4:** Solve Q with gradient descent by fixing U and V.

**5:** Solve V with gradient descent by fixing U and Q.

**6: end while**

**7:** Sorting U and V in descending order based on their absolute values and reporting the user or domain expert-defined top *M* biomarkers.

### 3.3 Convergence and complexity analysis

Theorem 1 *Algorithm 1 will monotonously decrease in each iteration, which reduces the computational complexity from* $O(np^{2})$  *to* $O(np_{k}^{2}K)$  *(*$p_{k}\ll p$, K≪p*).*

#### 3.3.1 Convergence analysis

According to optimization process, the sub-objectives for U, Q, and V are three convex sub-problems. This suggests that the convergence could be guaranteed when solving U, Q, and V alternatively. Based on bi-convex theory, the overall objective will converge as well as long as the sub-problems are convex. Through mathematical derivation, we can deduce that Ca-GPCA is bounded from below by zero. As a result, Algorithm 1 is guaranteed to converge to a local optimum.

#### 3.3.2 Complexity analysis

The main computational burden in the U-update arises from inverting $X^{T}X$, with complexity reduced to approximately $O(np^{2})$, where *n* is the number of subjects and *p* the number of features. In fast Ca-GPCA, this complexity further reduces to $O(np_{k}^{2}K)$, where $p_{k}$ is the dimension of the *k*th block. This is highly efficient since LD blocks are generally small (i.e. $p_{k}\ll p$, K≪p). Additionally, memory usage decreases, as fast Ca-GPCA only retains small SNP matrices during iteration.

## 4 Experiments on synthetic datasets and real neuroimaging genetics datasets

### 4.1 Experimental setup

#### 4.1.1 State-of-the-art methods

We compared Ca-GPCA to three related state-of-the-art approaches: SMCCA, AdaSMCCA, and RelPMDCCA, which were widely used to analyze multiomics brain imaging data (Witten and Tibshirani 2009, Hu *et al.* 2017, Rodosthenous *et al.* 2020), which were the most representative of the computational imaging genetics methods and could be reduced to various specific GP analytical methods.

#### 4.1.2 Evaluation criteria and parameter setting

Our performance evaluation relies on two key metrics: the identified feature subsets and canonical correlation coefficient (CCC). We employed the nested five-fold cross-validation to fine-tune parameters within the candidate set $10^{i}$ (i=−4,−3,−2,…,0,…,2,3,4). We selected parameters yielding the highest mean testing CCCs. Regarding the regression term for predicting patients’ diagnostic status, our model set the equal weighting of disease prediction and bidirectional association learning module. Our motivation for this choice was grounded in assigning equal optimization weight ensures that neither task is deprioritized, leading to a more predictive and interpretable multimodal fusion model.

### 4.2 Results on synthetic data

#### 4.2.1 Synthetic data source

We generated four simulation datasets with variations in sample size, dimensionality, and noise intensity. The first, second, and third datasets shared identical ground truths but differed in the intensity of noise, with the first dataset having the lowest signal-to-noise ratio (SNR), while the third dataset had the highest. The fourth dataset was characterized by a small sample size but high-dimensional features. The process for generating the simulation is described in detail as follows. Firstly, we generated a sparse vector $u\in R^{p\times 1}$ to simulate the effects of genetic markers and three sparse vectors $v_{1}\in R^{q_{1}\times 1}$, $v_{2}\in R^{q_{2}\times 1}$, $v_{3}\in R^{q_{3}\times 1}$ to simulate the important endophenotypes, i.e. imaging phenotypes, proteomic markers, and cognitive phenotypes. Then, using a latent vector μ, we created X by $x_{\ell ,i}∼N(\mu_{\ell }u_{i},\sigma_{x}\Sigma_{x})$, σ was the noise and we also designed group structures to simulate the linkage disequilibrium (LD) structures for genetic data. $Y_{j}$ was generated from ${\left(y_{\ell ,i}\right)}_{k}∼N(\mu_{\ell }v_{i,k},\sigma_{y}\Sigma_{y})$, and $\Sigma_{y}$ was the identity matrix.

#### 4.2.2 Better bi-multivariate correlation identification

Table 1 exhibits the testing CCCs of all methods on four simulated datasets simultaneously. For convenience, we denoted CCC between X and $Y_{i}$ as $\mathrm{CC}C_{i}$. In Data 1, 2, and 3, Ca*-*GPCA obtained higher CCC, suggesting that Ca*-*GPCA significantly and consistently outperform the start-of-the-art methods at different noise intensities. Meanwhile, by efficiently removing spurious causal association, the CCCs of Data 4 show that Ca*-*GPCA still outperformed benchmarks, thus Ca*-*GPCA is more capable of learning useful bi-multivariate associations under large-*p*-small-*n* settings. Of note, Ca*-*GPCA consistently held the smallest variance. This is probably because Ca*-*GPCA can take advantage of the stability of causal relationships and exploit the causal contribution of important features.

**Table 1.** Correlation results are presented on synthetic data from five-fold cross-validation, where CCC1-1 representing the CCC between X and $Y_{1}$, CCC1-2 between X and $Y_{2}$, and so forth.^a^

	Data 1			Data 2			Data 3			Data 4		
	CCC1-1	CCC1-2	CCC1-3	CCC1-1	CCC1-2	CCC1-3	CCC1-1	CCC1-2	CCC1-3	CCC1-1	CCC1-2	CCC1-3
	
	Testing CCCs											
SMCCA	0.96 ± 0.03	0.97 ± 0.02	0.96 ± 0.02	0.79 ± 0.03	0.87 ± 0.04	0.81 ± 0.04	0.62 ± 0.04	0.79 ± 0.06	0.66 ± 0.06	0.78 ± 0.04	0.88 ± 0.04	0.89 ± 0.04
AdaSMCCA	0.96 ± 0.03	0.96 ± 0.02	0.97 ± 0.02	0.79 ± 0.04	0.86 ± 0.03	0.80 ± 0.03	0.63 ± 0.03	0.78 ± 0.06	0.65 ± 0.05	0.80 ± 0.03	0.87 ± 0.03	0.89 ± 0.05
RelPMDCCA	0.96 ± 0.02	0.97 ± 0.03	0.96 ± 0.01	0.80 ± 0.03	0.86 ± 0.03	0.81 ± 0.03	0.62 ± 0.05	0.79 ± 0.04	0.67 ± 0.05	0.79 ± 0.04	0.89 ± 0.03	0.91 ± 0.02
Proposed	**0.98 ± 0.01**	**0.99 ± 0.00**	**0.98 ± 0.00**	**0.82 ± 0.01**	**0.89 ± 0.02**	**0.83 ± 0.02**	**0.66 ± 0.03**	**0.83 ± 0.02**	**0.69 ± 0.03**	**0.82 ± 0.01**	**0.92 ± 0.01**	**0.95 ± 0.00**

a Bold indicates the best result, underlined is second best.

#### 4.2.3 Better selected feature subsets

**Figure 3.**
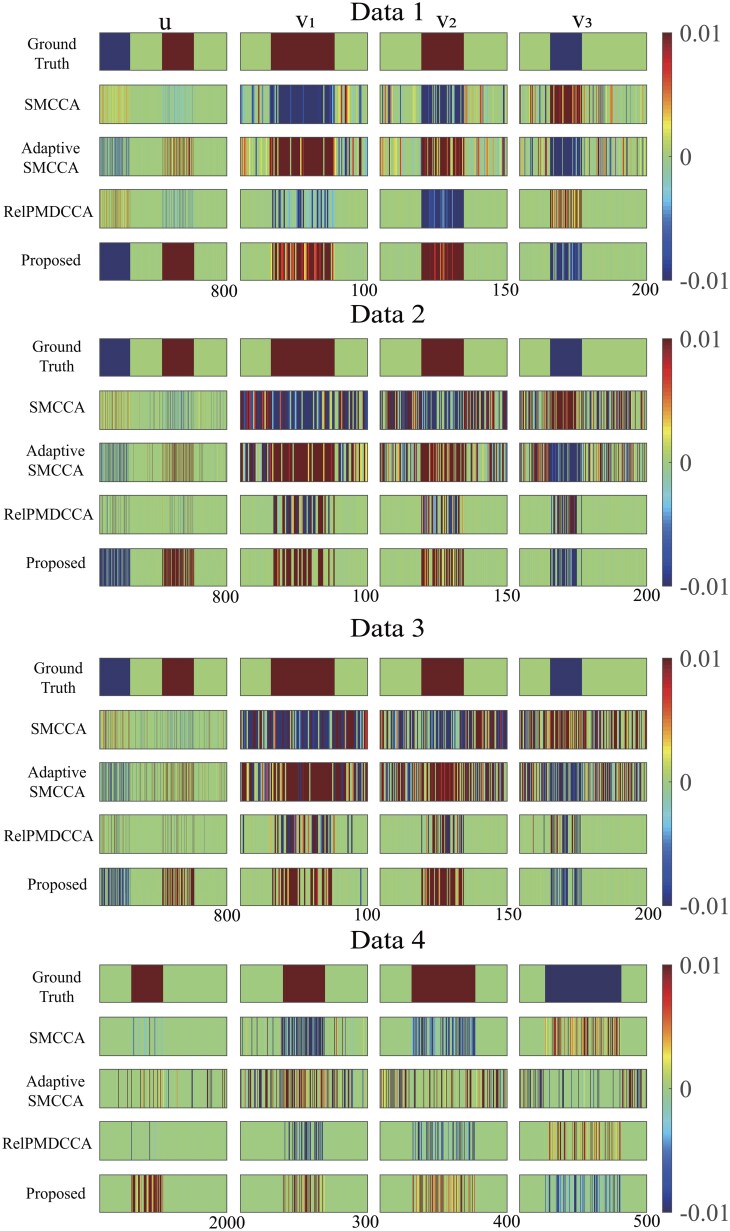
Canonical weights on synthetic data. Each row means: (1) ground truth; (2) SMCCA; (3) adaptive SMCCA; (4) RelPMDCCA; (5) proposed.

In addition to CCCs, detecting meaningful feature subsets was of significant importance. In Fig. 3, the feature selection results for Ca*-*GPCA and comparative methods were presented. Each dataset’s top row depicted the true signal of simulated data, while the subsequent rows represented the results of each method. The red and green colors represented the weights of the feature loci, where red indicated a positive weight and green indicates a negative weight. This color scheme was designed to evaluate whether different methods can successfully detect signals with varying effect directions. Our observations indicated that Ca*-*GPCA excelled in accurately identifying credible feature subsets aligned with the ground truths. In contrast, benchmark methods struggled to pinpoint the locations of true signals and yielded unsatisfactory performance in most cases. This discrepancy is likely due to their suffering from spurious causal correlations, which may lead to the omission of vital information.

### 4.3 Application to Alzheimer’s disease dataset

#### 4.3.1 Real neuroimaging genetics data source

Data used in the preparation of this article were obtained from the Alzheimer’s Disease Neuroimaging Initiative (ADNI) database (adni.loni.usc.edu). The ADNI was launched in 2003 as a public–private partnership, led by Principal Investigator Michael W. Weiner, MD. The primary goal of ADNI has been to test whether serial magnetic resonance imaging (MRI), positron emission tomography (PET), other biological markers, and clinical and neuropsychological assessment can be combined to measure the progression of mild cognitive impairment (MCI) and early Alzheimer’s disease (AD).

The dataset comprised 244 participants, including 42 healthy controls (HCs), 137 MCIs, and 65 ADs. First, voxel-based morphometry (VBM) was used to generate global gray matter (GM) density maps and extract local GM density values for target regions. Second, FreeSurfer-based automated parcellation defined volumetric and cortical thickness measures for regions of interest (ROIs) and extracted total intracranial volume (ICV). We selected 10 000 SNPs from ADNI and used the additive coding paradigm, i.e. the number of minor alleles, for SNPs. We also obtained other endophenotypes, i.e. proteomic, and cognition, to form the dataset.

### 4.4 Improved modeling performance

#### 4.4.1 Canonical correlation coefficient

**Figure 4.**
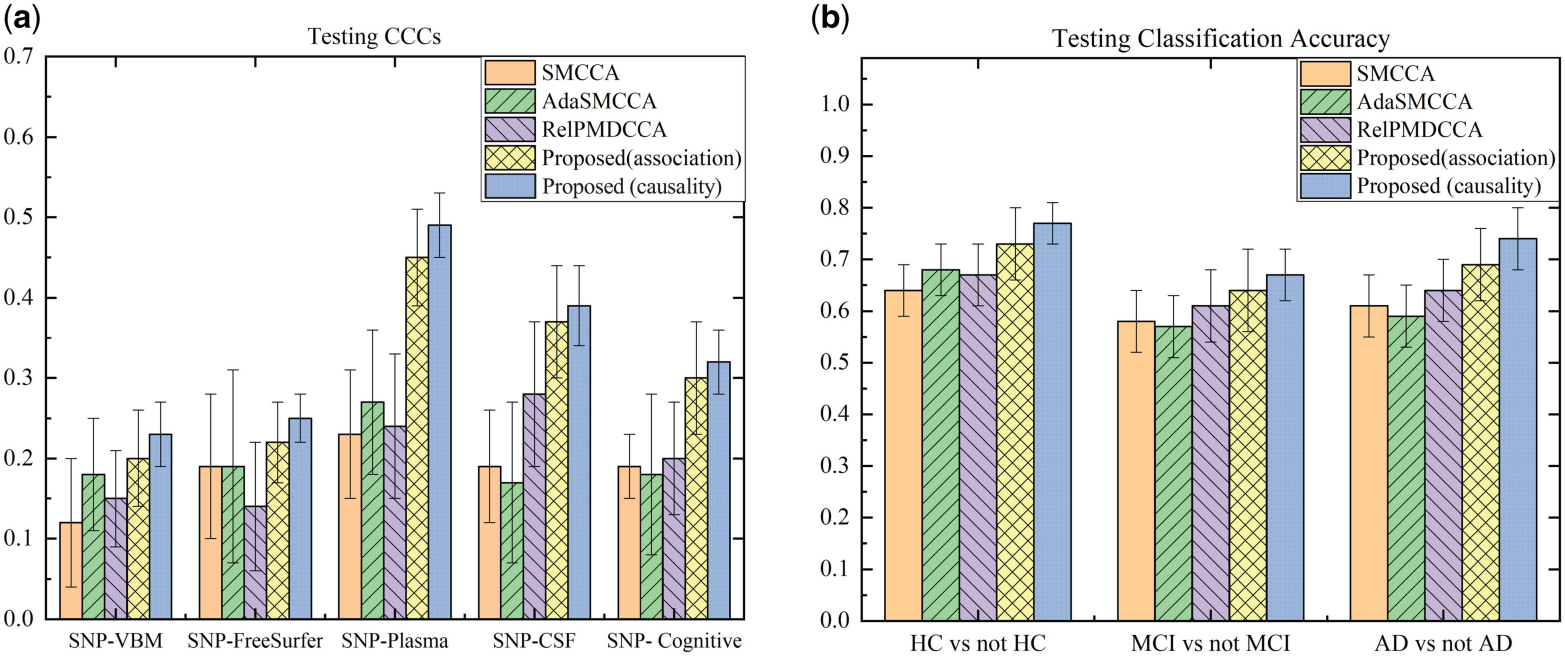
Comparison of CCCs and ACCs (mean ± std.) on ADNI dataset. Our model identifies causal relationships and exploits “causality” contribution for prediction. “association” means the parallel variable decorrelation module was removed from the model. (a) Testing CCCs (b) Testing Classification Accuracy.

Figure 4a illustrates the mean and standard deviation of CCCs for all methods. There were five GP correlation learning tasks, denoted as SNP-VBM, SNP-FreeSurfer, SNP-Plasma, SNP-CSF, and SNP-Cognitive respectively, where higher values indicated better performance. As anticipated, Ca*-*GPCA held the best average CCCs. Meanwhile, it can be observed that our model also outperformed comparison approaches with respect to CCC’s standard deviations, and thus we can safely attribute the significant improvement to the causal GP modeling strategy. Not surprisingly, the correlation-based bidirectional learning method did not work well in the GP correlation learning task, probably because they erroneously confounded direct associations (causal effects) and indirect associations (non-causal effects).

#### 4.4.2 Classification accuracy (ACC)

We further investigated the diagnosis performance of our method. Using the top 10 selected features, including SNP and endophenotypes, we used the LIBSVM software package to assess the method’s ability to distinguish HCs, MCIs, and AD patients. Figure 4b illustrates the mean and standard deviation of classification accuracy for all methods. Interestingly, our method obtained the best testing classification performance, including the highest classification accuracy and smallest standard error. This underscored that the top-ranked features of our algorithm had the best prediction ability for MCIs/ADs, owing to the reliable causal biomarker discovery strategy and prediction module.

### 4.5 Genetic marker explanation and biological implications

**Figure 5.**
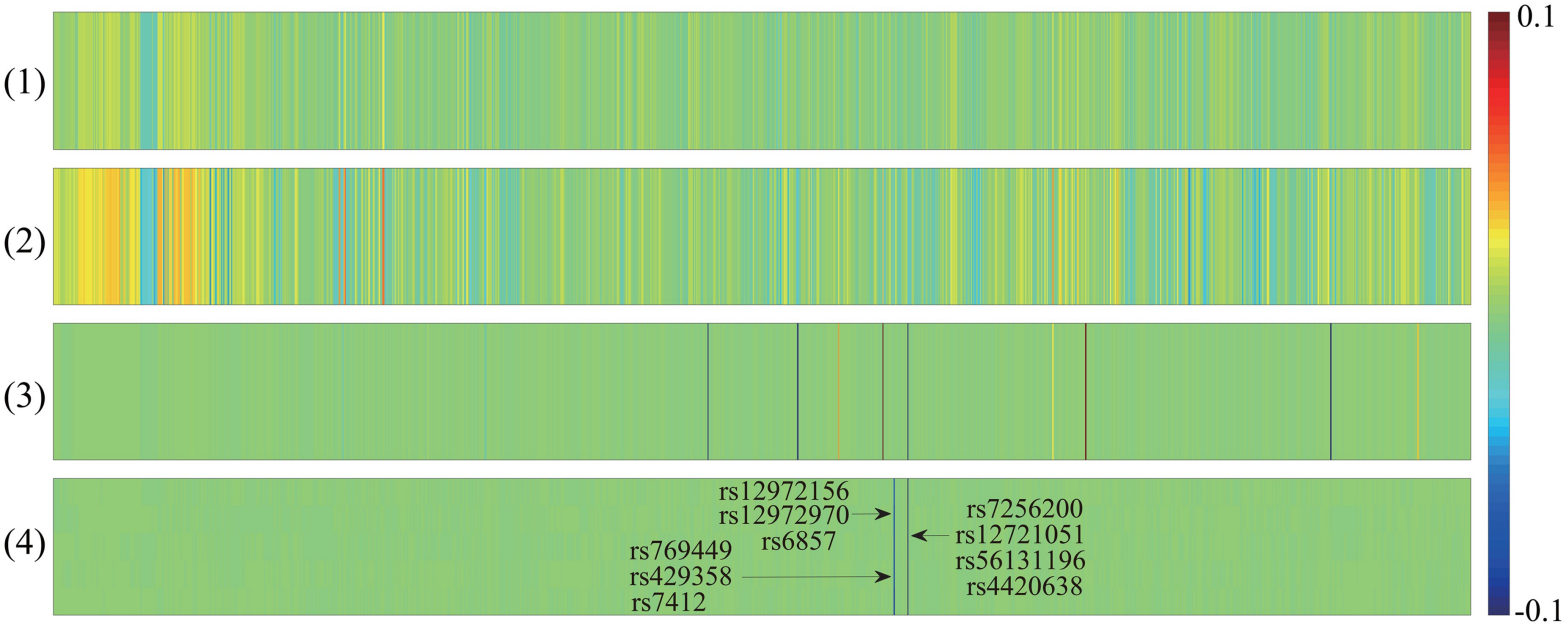
Canonical weights (mean value) of SNPs from five-fold cross-validation. Each row is a method: (1) SMCCA; (2) adaptive SMCCA; (3) RelPMDCCA; (4) Ca*-*GPCA.

Figure 5 and Table 2 present the top 10 SNPs. Surprisingly, multiple AD-related risk loci were successfully identified by Ca*-*GPCA, including rs429358 and rs7412 (located in *APOE*), rs769449, rs4420638, rs56131196, rs12721051, and rs7256200 (*APOC*1), rs6857 (*TOMM*40), and rs12972970, rs12972156 (*PVRL*2) (Zhang *et al.* 2022a). Another interesting observation was that rs429358 exhibited the highest negative weight, while the rs7412 had the highest positive weight, showing an opposite effect. These findings aligned with the existing findings. That is, the minor homozygote of rs429358 had a high risk of AD, while the major homozygote of rs7412 resulted in a high risk of AD. In contrast, competitors reported numerous irrelevant signals that could potentially mislead subsequent analyses. All these results suggested that our method could identify genetic risk factors accurately and comprehensively due to the introduction of causality and interpretable regularized modules on bidirectional association learning.

**Table 2.** Top 10 loci of each method based on mean canonical weights.^a^

SMCCA		AdaSMCCA		RelPMDCCA		Ca*-*GPCA	
SNP_ID	Weight	SNP_ID	Weight	SNP_ID	Weight	SNP_ID	Weight
rs140627212	−0.0275	rs2599457	0.0598	rs6857	−0.0802	rs429358	−0.6933
rs117998908	−0.0275	rs2682567	0.0598	rs7412	0.0801	rs6857	−0.1656
rs79572806	−0.0271	rs12980473	0.0589	rs113990109	0.0637	rs4420638	−0.0858
rs55936413	−0.0268	rs34536923	0.0580	rs147901416	−0.0617	rs56131196	−0.0416
rs56256456	−0.0268	rs3213332	0.0567	rs79074020	0.0447	rs12721051	−0.0272
rs111463774	−0.0268	rs35681564	0.0549	rs3112439	−0.0435	rs7256200	−0.0035
rs56227398	−0.0268	rs2682562	0.0546	rs117565628	−0.0397	rs7412	0.0027
rs62118221	−0.0268	rs2682560	0.0546	rs117184667	−0.0390	rs769449	−0.0013
rs2599457	−0.0265	rs2599464	0.0546	rs146160549	−0.0377	rs12972970	−0.0006
rs2682567	−0.0265	rs25484	0.0546	rs13382123	0.0362	rs12972156	−0.0005

a The weight value showed the effect of each SNP.

### 4.6 Phenotype explanation and biological implications

**Figure 6.**
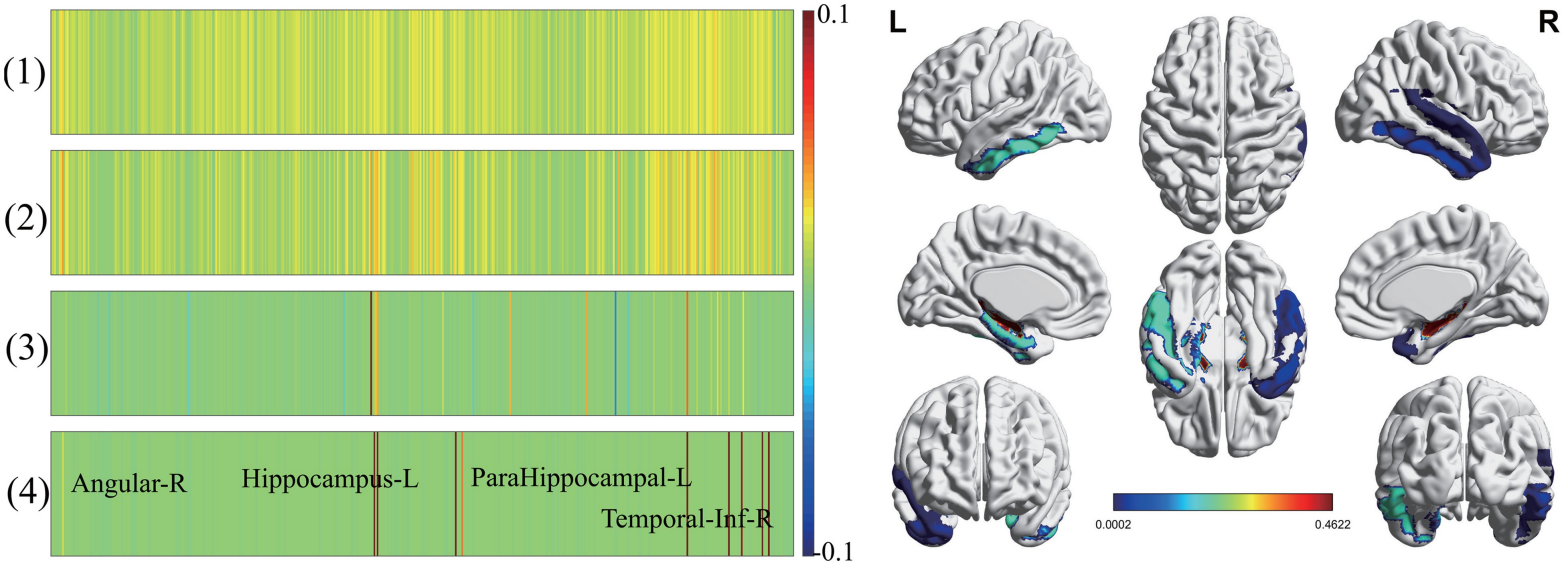
Visualization of identified brain imaging phenotype (left), each row is a method: (1) SMCCA; (2) adaptive SMCCA; (3) RelPMDCCA; (4) Ca*-*GPCA. We also mapped the canonical weights into a real brain template (right).

Identifying abnormal intermediate phenotypes affected by AD held great promise for computer-aided diagnosis. We presented the top selected imaging phenotypes identified by Ca-GPCA in Fig. 6. Notably, Ca*-*GPCA successfully identified crucial brain regions, including right angular, left hippocampus, and so forth (Shen *et al.* 2010). According to literature research, the angular gyrus could improve cognitive function in AD patients, the hippocampus area was associated with memory, and both of them were significant indicators for diagnosing AD. In addition, extended experiments on other phenotypes also demonstrated the superiority of our framework. For example, plasma-derived proteomic markers such as ApoE, BNP, CSF-derived proteomic markers FGF-4, VEGF, and cognitive markers of ADAS, MMSE, and so forth, had been verified to be related to AD. These results highlighted the superior performance of Ca*-*GPCA in identifying credible AD-affected phenotypes.

### 4.7 GP-correlated detection

**Figure 7.**
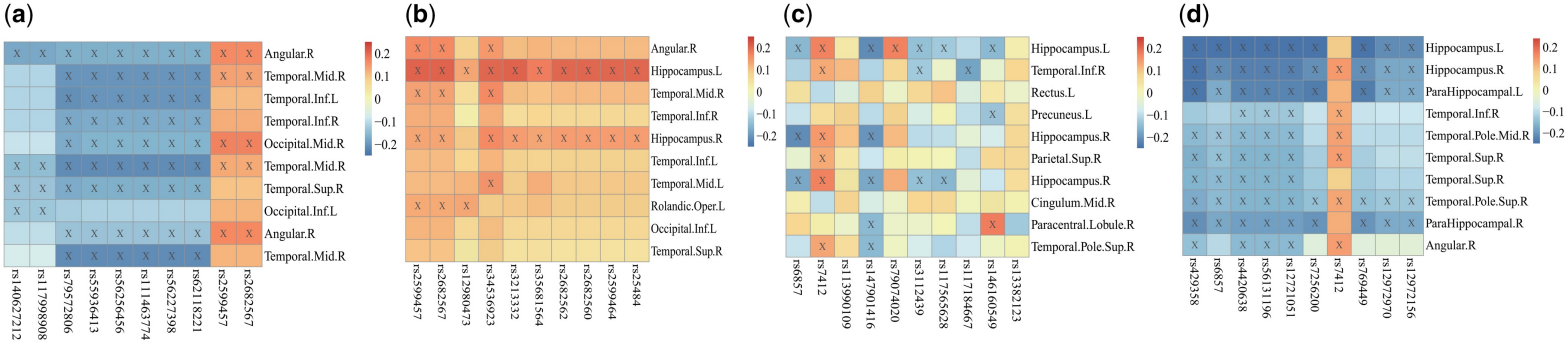
Pairwise correlation between the identified SNPs and intermediate phenotypes. The symbol “×” indicates that a genotype–phenotype pair reaches statistical significance (*P* > .05).

To further illustrate the biological effect, we performed a GP correlation analysis of each method (a–c, baseline; d, proposed). As shown in Fig. 7, most pairwise correlations of GP were significant. For instance, rs429358 and rs7412 exhibited significant associations with right angular, supporting a high heritability of angular abnormality. Moreover, the correlation pattern of rs7412 was opposite to that of rs429358, which was consistent with existing research (Zhou *et al.* 2019). This again verified the value of finding causal effects by Ca*-*GPCA. Furthermore, a two-way ANOVA was applied to investigate GP interactions on the diagnostic phenotype, i.e. interactions between rs429358 and right angular, and between rs7412 and right angular. Of particular interest, these results revealed several significant findings. First, the main effects of rs429358 (*P* = $5.20\times 10^{-10}$*) and right angular (*P* = $4.69\times 10^{-6}$*) were statistically significant, and the interaction between them was also significant, i.e. *P* = $1.83\times 10^{-2}$*. In addition, the main effects of rs7412 (*P* = $3.60\times 10^{-6}$*) and angular right (*P* = $3.08\times 10^{-6}$*) were statistically significant, and rs7412 by right angular interaction showed a significant effect too, i.e. *P* = $2.24\times 10^{-3}$* (* indicated reaching the significance level). However, such interesting results were omitted by baselines, which again suggested the value of distinguishing causal effects from spurious indirect correlations. This further underscored the capability of Ca*-*GPCA in identifying causal relationships and meaningful genetic and phenotypes for AD.

### 4.8 Ablation study

To investigate the effectiveness of Ca*-*GPCA, we conducted a series of ablation studies. First, we ran ablation experiments to investigate the impact of each component on GP correlation and HC/MCI/AD classification tasks on the ADNI dataset. Next, we comprehensively analyzed the influence of hyper-parameters of Ca*-*GPCA.

#### 4.8.1 Effect of parallel causal decorrelation module

As shown in Table 3 and Fig. 4, our method outperformed baselines, including correlation-based approaches, achieving the highest average MCCC and MACC with the lowest standard error. This highlighted the effectiveness of the parallel causal decorrelation module in enhancing correlation and biomarker identification. Additionally, Fig. 4b demonstrates that Ca-GPCA’s top-ranked features exhibited the best predictive performance, achieving the highest MACC with the smallest standard error, emphasizing the importance of eliminating indirect correlations for causal biomarker identification. Moreover, an additional experiment showed that the divide-and-conquer strategy reduces computation time by a 100-fold (from 5102 to 57 s) without compromising performance.

**Table 3.** Ablation studies on different modules.^a^

Id	$L_{\text{sparsity}}$			$L_{\text{causal}}$	MCCC↑	MACC↑
	$\ell_{1}$ -norm	$\ell_{2,1}$ -norm	${\text{FGL}}_{2,1}$ -norm			
(a)	×	×	×	×	0.27 ± 0.07	0.63 ± 0.09
(b)	√	×	×	×	0.29 ± 0.06	0.66 ± 0.08
(c)	√	√	×	×	0.30 ± 0.06	0.68 ± 0.07
(d)	√	√	√	×	0.31 ± 0.06	0.69 ± 0.07
(e)	√	√	√	√	**0.34** **±** **0.04**	**0.73** **±** **0.05**

a $L_{\text{causal}}$ denotes the parallel causal decorrelation module. $L_{\text{sparsity}}$ represents sparse variable regularizer modules. MCCC indicates mean testing correlation coefficients for all genotype intermediate phenotypes. MACC indicates mean testing classification accuracy for all diagnostic groups. Bold indicates the best result, underlined is second best. ↑ means higher is better.

#### 4.8.2 Effect of sparse variable regularizer modules

In Table 3, we notice that the performance without $L_{\text{sparsity}}$ is worse, indicating the necessity of introducing sparse variable regularizer modules into predictive tasks, which could enhance Ca-GPCA’s prediction ability.

#### 4.8.3 Effect of the joint group and individual feature selection

Table 3 exhibits ablation results for different penalties. The highest MCCC and MACC were obtained when all penalties were included, and the worst ones were obtained when excluding ${\text{FGL}}_{2,1}$-norm. These findings could be attributed to the oligogenic or polygenic characteristic of AD. Thus, our hybrid penalty could not only enable a diverse and flexible feature selection but also help AD diagnosis.

#### 4.8.4 Effect of the disease diagnosis module

An addition ablation study on the disease diagnosis module showed that including it improved MACC by 4%, which could lead to earlier detection of potential health issues, enabling timely interventions and ultimately improving patient outcomes. This confirmed its role in enhancing diagnostic accuracy and causal effect identification.

#### 4.8.5 Influence of hyperparameters

We conducted ablation experiments to assess the impact of empirically chosen hyper-parameters. The margin η served as a tradeoff, balancing bidirectional association learning and parallel causal variable decorrelation. A larger η enhanced GP correlations by eliminating indirect associations, whereas a smaller η increased the risk of identifying spurious biomarkers. If too small, Ca-GPCA yielded suboptimal, hard-to-interpret results; if too large, it over-penalized outcomes. After performing grid search, our analysis identified η=0.1 as the optimal value, which provided the best predictive performance.

## 5 Discussion

Our findings demonstrated that causality-aware modeling can improve the reliability and interpretability of biomarker discovery for brain disorders. Unlike traditional association-based methods, Ca-GPCA explicitly disentangled spurious correlations and highlighted clinically relevant genetic factors. This dual focus on pathogenesis and diagnosis supports its translational potential in precision neuromedicine. Moreover, the framework’s computational efficiency enables future integration with federated learning, making it well-suited for privacy-sensitive settings such as multi-center genomic studies. Together, these advantages position Ca-GPCA as a promising tool for secure and scalable biomarker discovery.

## 6 Conclusion

We proposed Ca*-*GPCA to extract causal relationships between brain endophenotypes and genetic variations while concurrently performing disease diagnosis to detect disease-related biomarkers. Additionally, we extended the current task to a chromosome-wide setting and produced strong baseline results. Furthermore, our method exhibited reasonable performance in extensive experiments, confirming its effectiveness and value. To validate the pathophysiological significance of the identified multimodal biomarkers, we conducted follow-up analyses, including ANOVA, multimodal correlation, and disease diagnosis. These analyses confirmed the relevance of the identified risk factors. In future endeavors, we are expanding our dataset with diverse cohorts to enhance generalizability. Future studies will validate our findings across multi-ethnic populations to ensure broader applicability.

## Supplementary Material

btaf257_Supplementary_Data

## Data Availability

Data used in the preparation of this article were obtained from ADNI database (adni.loni.usc.edu).
