# Supplementary material for: Trustworthy causal biomarker discovery: a multiomics brain imaging genetics-based approach
Source: Bioinformatics. 2025 Jul 15;41(Suppl 1):i227–36. doi: 10.1093/bioinformatics/btaf257 (PMC12261482; doi:10.1093/bioinformatics/btaf257)
Supplement: btaf257_Supplementary_Data [file btaf257_supplementary_data.pdf]

Appendix / supplemental material for "**Trustworthy Causal Biomarker Discovery: A Multiomics Brain Imaging Genetics Oriented Approach**"

A. Efficient Optimization Algorithm

To solve the proposed Ca-GPCA, we first present its Lagrangian form as follows:

$$\begin{aligned} L(\mathbf{U},\mathbf{Q},\mathbf{V}) = &\sum_{f=1}^{\mathrm{F}} -\mathbf{u}_f^\top \mathbf{X}^\top \operatorname{diag}(\mathbf{Q})\mathbf{Y}_f\mathbf{v}_f + \psi\left(\mathbf{v}_f;\mathbf{Y}_f,\mathbf{z}\right) \\ &+\eta\sum_{k=1}^{K}\sum_{i,j\in\Pi_k}\left\|\mathbb{E}\left(\mathbf{X}_i^{\mathrm{T}}\Sigma_{\mathbf{Q}}\mathbf{X}_j\right)-\mathbb{E}\left(\mathbf{X}_i^{\mathrm{T}}\mathbf{Q}\right)\mathbb{E}\left(\mathbf{X}_j^{\mathrm{T}}\mathbf{Q}\right)\right\|_2^2 \\ &+\Omega(\mathbf{U})+\Omega(\mathbf{Q})+\Omega(\mathbf{V})+\gamma_1\sum_{f=1}^{F}\|\mathbf{X}\mathbf{u}_f\|_2^2+\gamma_2\sum_{f=1}^{F}\|\mathbf{Y}_f\mathbf{v}_f\|_2^2 \end{aligned} \tag{1}$$

This objective is multi-convex and smooth, and thus, we solve $\mathbf{U}$ via fixing $\mathbf{Q}$, $\mathbf{V}$. We take the derivative with respect to Eq. (1) $\mathbf{U}$, and further obtain closed-form updating rule:

$$\mathbf{U}=\left(\lambda_{\mathbf{u}_1}\mathbf{D}_{\mathbf{u}_1}+\lambda_{\mathbf{u}_2}\mathbf{D}_{\mathbf{u}_2}+\lambda_{\mathbf{u}_3}\mathbf{D}_{\mathbf{u}_3}+\gamma_1'\mathbf{X}^\top\mathbf{X}\right)^{-1}\mathbf{X}^\top\mathbb{Y} \tag{2}$$

We define $\mathbb{Y}=[\operatorname{diag}(\mathbf{Q})\mathbf{Y}_1\mathbf{v}_1,\cdots,\operatorname{diag}(\mathbf{Q})\mathbf{Y}_F\mathbf{v}_F]$. To meet the equality constraints, we scale $\mathbf{u}$ as $\mathbf{u}_f=\frac{\mathbf{u}_f}{\|\mathbf{X}\mathbf{u}_f\|_2}$, $\forall f=1,2,\cdots,F$. $\mathbf{D}_{\mathbf{u}_1}$, $\mathbf{D}_{\mathbf{u}_2}$ and $\mathbf{D}_{\mathbf{u}_3}$ represent diagonal matrices, and all of them are sub-gradients.* In addition, γ_1' is a non-negative tuning parameters.

Following analogous procedures, we could obtain the loss concerning $\mathbf{Q}$ as follows:

$$\begin{aligned} J(\mathbf{Q}) = &\sum_{f=1}^{\mathrm{F}} -\mathbf{u}_f^\top \mathbf{X}^\top \operatorname{diag}(\mathbf{Q})\mathbf{Y}_f\mathbf{v}_f \\ &+\eta\sum_{k=1}^{K}\sum_{i,j\in\Pi_k}\left\|\mathbb{E}\left(\mathbf{X}_i^{\mathrm{T}}\Sigma_{\mathbf{Q}}\mathbf{X}_j\right)-\mathbb{E}\left(\mathbf{X}_i^{\mathrm{T}}\mathbf{Q}\right)\mathbb{E}\left(\mathbf{X}_j^{\mathrm{T}}\mathbf{Q}\right)\right\|_2^2 \\ &+\lambda_{Q_1}\|\mathbf{Q}\|_2^2+\lambda_{Q_2}\left(\sum_{k=1}^{n}\mathbf{Q}_k-n\right)^2. \end{aligned} \tag{3}$$

Then we can iteratively update $\mathbf{Q}$ with gradient descent by fixing $\mathbf{U}$ and $\mathbf{V}$: *i.e.*, $\mathbf{Q}=\mathbf{Q}-\alpha\cdot\nabla_{\mathbf{Q}}J(\mathbf{Q})$. Similarly, the sub-objective with respect to $\mathbf{V}$ is:

$$\begin{aligned} J(\mathbf{V}) = &\sum_{f=1}^{\mathrm{F}} -\mathbf{u}_f^\top \mathbf{X}^\top \operatorname{diag}(\mathbf{Q})\mathbf{Y}_f\mathbf{v}_f + \psi\left(\mathbf{v}_f;\mathbf{Y}_f,\mathbf{z}\right) \\ &+\lambda_v\|\ \mathbf{V}\|_{1,1}+\gamma_2\sum_{f=1}^{F}\|\mathbf{Y}_f\mathbf{v}_f\|_2^2. \end{aligned} \tag{4}$$

*$\mathbf{D}_{\mathbf{u}_1}\mathbf{U}$, $\mathbf{D}_{\mathbf{u}_2}\mathbf{U}$ and $\mathbf{D}_{\mathbf{u}_3}\mathbf{U}$ are sub-gradients of $\|\mathbf{U}\|_{\mathrm{FGL}_{2,1}}$, $\|\mathbf{U}\|_{2,1}$ and $\|\mathbf{U}\|_{1,1}$. $\mathbf{D}_{\mathbf{u}_1}$ is a diagonal matrixwhere the i-th diagonal block is $\frac{1}{2\sqrt{\|\mathbf{u}^{i-1}\|_2^2+\|\mathbf{u}^i\|_2^2}}+\frac{1}{2\sqrt{\|\mathbf{u}^i\|_2^2+\|\mathbf{u}^{i+1}\|_2^2}}$; $\mathbf{D}_{\mathbf{u}_2}$ is another diagonal matrix x where the i-th diagonalblock is $\frac{1}{2\|\mathbf{u}^i\|_2}$, $\mathbf{D}_{\mathbf{u}_3}$ is also a diagonal matrix with diagonal entries being $\frac{1}{2|\mathbf{u}|}$.

Therefore, $\mathbf{V}$ can be obtained though the gradient descent method with $\mathbf{U}$ and $\mathbf{Q}$ fixed as constants: *i.e.*, $\mathbf{V} = \mathbf{V} - \alpha \cdot \nabla_{\mathbf{V}} J(\mathbf{V})$. To satisfy the equality constraints, $\mathbf{V}$ is further scaled by $\mathbf{v}_f = \frac{\mathbf{v}_f}{\|\mathbf{Y}_f \mathbf{v}_f\|_2}, \quad \forall f = 1, 2, \cdots, F$.

B. Theoretical Analysis

B.1. Analysis on fast Ca-GPCA

Directly applying Ca-GPCA to genome-wide analysis posed computational challenges, primarily due to the computationally intensive covariances involved, making it exceptionally burdensome. Therefore, we devised a fast Ca-GPCA optimization algorithm, which can significantly alleviate the computational burden while maintaining the model's performance. This improvement was substantiated by the following theorem.

Theorem 1. *Considering* $\mathbf{X}^\top \mathbf{X}$ *is a block diagonal matrix, we could obtain the closed-form rule of* $\mathbf{U}$*, i.e.,* $\mathbf{U} = \oplus_{k=1}^{K} \mathbf{U}^k$*, in the spirit of divide and conquer:*

$$\mathbf{U}^{\mathrm{k}} = \left(\lambda_{u_1} \mathbf{D}_{u_1 \mathrm{gk}} + \lambda_{u_2} \mathbf{D}_{u_2 \mathrm{gk}} + \lambda_{u_3} \mathbf{D}_{u_3 \mathrm{gk}} + \gamma_1' \left(\mathbf{X}^\top \mathbf{X}\right)_{\mathrm{gk}}\right)^{-1} \mathbf{X}_{\mathrm{gk}}^\top \mathbb{Y} \tag{5}$$

where $\oplus$ represents the matrix concatenation operation.

Proof: According to the LD structures of SNPs, we partitioned the large genotype matrix into smaller submatrices, with each dimension being equivalent to the corresponding LD block size. Let $\mathbf{X} = (x_1, \cdots, x_k, \cdots, x_K)$ be the genotype matrix, with k being the index of the k-th block, the covariance matrix $\mathbf{X}^\top \mathbf{X}$ can be represented as:

$$\mathbf{X}^\top \mathbf{X} = \begin{bmatrix} \ddots & & \\ & \left(\mathbf{x}^\top \mathbf{x}\right)_{g_k} & \\ & & \ddots \end{bmatrix}. \tag{6}$$

Since $\mathbf{D}_{u_1}$, $\mathbf{D}_{u_2}$ and $\mathbf{D}_{u_3}$ were diagonal matrices, they were diagonal separable. Thus, we could obtain a closed-form updating rule for $\mathbf{U}$:

$$\mathbf{U} = \left(\begin{bmatrix} \ddots & & \\ & \lambda_{u_1} \mathbf{D}_{u_1 \mathrm{gk}} + \lambda_{u_2} \mathbf{D}_{u_2 \mathrm{gk}} + \lambda_{u_3} \mathbf{D}_{u_3 \mathrm{gk}} + \gamma_1' \mathbf{x}_{g_k}^\top \mathbf{x}_{g_k} & \\ & & \ddots \end{bmatrix}\right)^{-1} \begin{bmatrix} \vdots \\ \mathbf{X}_{g_k}^\top \\ \vdots \end{bmatrix} \mathbb{Y} \tag{7}$$

On this account, we had the following formula:

$$\mathbf{U} = \begin{bmatrix} \vdots \\ \left(\lambda_{u_1}\mathbf{D}_{u_1\mathrm{gk}} + \lambda_{u_2}\mathbf{D}_{u_2\mathrm{gk}} + \lambda_{u_3}\mathbf{D}_{u_3\mathrm{gk}} + \hat{\gamma}_1' \left(\mathbf{X}^\top\mathbf{X}\right)_{g_k}\right)^{-1} \mathbf{X}_{g_k}^\top \mathbb{Y} \\ \vdots \end{bmatrix} = \oplus_{k=1}^{K}\mathbf{U}^k. \tag{8}$$

Therefore, the proof completes. Notably, the SNP information was predominantly represented by a set of block matrices along the diagonal. This is because that the off-block-diagonal elements of the covariance $\mathbf{X}^\top\mathbf{X}$ carries almost no information due to the genetic characteristic of the human genome. Thus, this approach was efficient and could successfully preserve essential genetic information within LD blocks. Furthermore, the size of LD blocks of human genome was relatively small, with an overwhelming majority (95.15%) containing fewer than 50 SNPs. Therefore, we established the block structure based on LD patterns, mitigating computational complexities while upholding model performance, and the memory requirement was also decreased, as fast Ca-GPCA only stored small SNP matrices during the iteration.

B.2. Convergence Analysis of Ca-GPCA

Theorem 2. *Algorithm 1 will monotonously decrease in each iteration.*

Proof: The proof can be partitioned into two parts: the first part established convergence for $\mathbf{U}$, while the second part addressed convergence for $\mathbf{Q}$ and $\mathbf{V}$.

Part 1: The estimation for the t-th iteration was denoted as $\left\{\mathbf{U}^{(\mathrm{t})},\ \mathbf{Q}^{(\mathrm{t})},\ \mathbf{V}^{(\mathrm{t})}\right\}$. For clarity, we defined the sub-objective of Ca-GPCA concerning $\mathbf{U}$ as follows:

$$\mathcal{F}(\mathbf{U}) \stackrel{\mathrm{def}}{=} \sum_{f=1}^{\mathrm{F}} -\mathbf{u}_f^\top \mathbf{X}^\top \operatorname{diag}(\mathbf{Q})\mathbf{Y}_f \mathbf{v}_f + \lambda_{\mathbf{u_1}} \|\mathbf{U}\|_{\mathrm{FGL}_{2,1}} + \lambda_{\mathbf{u_2}} \|\mathbf{U}\|_{2,1} + \lambda_{\mathbf{u_3}} \|\mathbf{U}\|_{1,1}. \tag{9}$$

Now we introduced an auxiliary function:

$$\begin{aligned}\mathcal{G}(\mathbf{U}) = &\sum_{f=1}^{\mathrm{F}} -\mathbf{u}_f^\top \mathbf{X}^\top \operatorname{diag}(\mathbf{Q})\mathbf{Y}_f \mathbf{v}_f + \lambda_{\mathbf{u_1}} \\ &\sum_{i=1}^{p-1}\left(\frac{\|\mathbf{u}^i\|_2^2 + \|\mathbf{u}^{i+1}\|_2^2}{2\sqrt{\|(\mathbf{u}^i)^{(t)}\|_2^2 + \|(\mathbf{u}^{i+1})^{(t)}\|_2^2}} + \frac{\sqrt{\|(\mathbf{u}^i)^{(t)}\|_2^2 + \|(\mathbf{u}^{i+1})^{(t)}\|_2^2}}{2}\right) + \lambda_{\mathbf{u_2}} \\ &\sum_{i}^{p}\left(\frac{\|\mathbf{u}^i\|_2^2}{2\left\|(\mathbf{u}^i)^{(t)}\right\|_2} + \frac{\left\|(\mathbf{u}^i)^{(t)}\right\|_2}{2}\right) + \lambda_{\mathbf{u_3}} \sum_{f=1}^{F}\sum_{i=1}^{p}\left(\frac{\mathrm{u}_{fi}^2}{2\left|\mathbf{u}_{fi}^{(t)}\right|} + \frac{\left|\mathbf{u}_{fi}^{(t)}\right|}{2}\right)\end{aligned} \tag{10}$$

$\mathcal{G}(\mathbf{U})$ can be equivalently reformulated as follows:

$$\begin{aligned}\mathcal{G}(\mathbf{U}) = &\sum_{f=1}^{\mathrm{F}} -\mathbf{u}_f^\top \mathbf{X}^\top \operatorname{diag}(\mathbf{Q})\mathbf{Y}_f \mathbf{v}_f + \frac{\lambda_{u_1}}{2}\left(\mathbf{U}^\top\mathbf{D}_{u_1}\mathbf{U} + \mathbf{U}^{(t)\top}\mathbf{D}_{u_1}\mathbf{U}^{(t)}\right) \\ &+ \frac{\lambda_{u_2}}{2}\left(\mathbf{U}^\top\mathbf{D}_{u_2}\mathbf{U} + \mathbf{U}^{(t)\top}\mathbf{D}_{u_2}\mathbf{U}^{(t)}\right) + \frac{\lambda_{u_3}}{2}\left(\mathbf{U}^\top\mathbf{D}_{u_3}\mathbf{U} + \mathbf{U}^{(t)\top}\mathbf{D}_{u_3}\mathbf{U}^{(t)}\right)\end{aligned} \tag{11}$$

where $\mathbf{D}_{\mathbf{u}_1}$, $\mathbf{D}_{\mathbf{u}_2}$ and $\mathbf{D}_{\mathbf{u}_3}$ represented diagonal matrices. Then we can verify:

$$\mathcal{G}\left(\mathbf{U}^{(t)}\right) = \mathcal{F}\left(\mathbf{U}^{(t)}\right), \text{ and } \mathcal{F}\left(\mathbf{U}\right) \leq \mathcal{G}\left(\mathbf{U}\right). \tag{12}$$

Furthermore, $\mathcal{G}\left(\mathbf{U}\right)$ represented a convex quadratic function, known for its smoothness and differentiability at all points, and satisfied the following condition:

$$\mathcal{G}\left(\mathbf{U}^{(t+1)}\right) \leq \mathcal{G}\left(\mathbf{U}^{(t)}\right). \tag{13}$$

Combining the aforementioned equations, we can deduce the following result:

$$\mathcal{F}\left(\mathbf{U}^{(t+1)}\right) \leq \mathcal{G}\left(\mathbf{U}^{(t+1)}\right) \leq \mathcal{G}\left(\mathbf{U}^{(t)}\right) = \mathcal{F}\left(\mathbf{U}^{(t)}\right). \tag{14}$$

The scaling steps employed have no impact on this conclusion, and thus affirming the completion of the first phase of the proof.

Part 2: Likewise, the same conclusion can be readily derived for the remaining variables, *i.e*, $\mathbf{Q}$ and $\mathbf{V}$.

Further, denoting the original objective of our method as $\mathcal{L}(\mathbf{U}, \mathbf{Q}, \mathbf{V})$, we can express it as follows:

$$\begin{aligned} & L\left(\mathbf{U}^{(t+1)}, \mathbf{Q}^{(t+1)}, \mathbf{V}^{(t+1)}\right) \\ \leq\ & L\left(\mathbf{U}^{(t+1)}, \mathbf{Q}^{(t+1)}, \mathbf{V}^{(t)}\right) \leq L\left(\mathbf{U}^{(t+1)}, \mathbf{Q}^{(t)}, \mathbf{V}^{(t)}\right) \\ \leq\ & L\left(\mathbf{U}^{(t)}, \mathbf{Q}^{(t)}, \mathbf{V}^{(t)}\right) \end{aligned} \tag{15}$$

Through mathematical derivation, a lower bound of zero can be established for the objective function of Ca-GPCA, ensuring the convergence of Algorithm 1 to a local optimum. To improve efficiency in practical implementation, we integrated early stopping conditions, *i.e.*, $\max\max|\mathbf{U}^{(t+1)} - \mathbf{U}^{(t)}| \leq \epsilon$, $\max\max|\mathbf{Q}^{(t+1)} - \mathbf{Q}^{(t)}| \leq \epsilon$ and $\max\max|\mathbf{V}^{(t+1)} - \mathbf{V}^{(t)}| \leq \epsilon$, where the tolerance error ϵ was set to 10^{-5}.

C. Experiments on Synthetic Datasets and Real Neuroimaging Genetics Datasets

Synthetic Data Source: We generated four simulation datasets with variations in sample size, dimensionality and noise intensity. The first, second and third datasets shared identical ground truths but differed in the intensity of noise, with the first dataset having the lowest signal-to-noise ratio (SNR), while the third dataset had the highest. The fourth dataset was characterized by a small sample size but high-dimensional features. The process for generating the simulation is described in detail as follows. Firstly, we generated a sparse vector $\mathbf{u} \in \mathcal{R}^{p\times 1}$ to simulate the effects of genetic markers and three sparse vectors $\mathbf{v_1} \in \mathcal{R}^{q_1\times 1}$,$\mathbf{v_2} \in \mathcal{R}^{q_2\times 1}$,$\mathbf{v_3} \in \mathcal{R}^{q_3\times 1}$ to simulate the important endophenotypes, *i.e.*, imaging phenotypes, proteomic markers and cognitive phenotypes. Then, using a latent vector μ, we created $\mathbf{X}$ by $x_{\ell,i} \sim N(\mu_\ell u_i, \sigma_x \Sigma_x)$, σ was the noise and we also designed group structures to simulate the linkage disequilibrium (LD) structures for genetic data. $\mathbf{Y}_j$ was generated from $(y_{\ell,i})_k \sim N(\mu_\ell v_{i,k}, \sigma_y \Sigma_y)$, and Σ_y was the identity matrix.

	HC	MCI	AD
Num	42	137	65
Gender (M/F, %)	52.38/47.62	69.34/30.66	55.38/44.62
Handedness (R/L, %)	90.48/9.52	92.70/7.30	98.46/1.54
Age (mean±std)	75.40±5.80	74.13±7.22	74.75±7.67
Education (mean±std)	15.88±2.77	16.03±2.98	15.12±3.05

Table 1: Participant characteristics.

Real Neuroimaging Genetics Data Source: The SNPs, endophenotypes including neuroimaging phenotypes, proteomic analytes, and cognitive phenotypes utilized in this study were obtained from the Alzheimer's Disease Neuroimaging Initiative (ADNI) database at adni.loni.usc.edu. The main aim of the ADNI database was to explore the viability of integrating serial magnetic resonance imaging (MRI), positron emission tomography (PET), additional biological markers, along with clinical and neuropsychological assessments, to assess the progression of mild cognitive impairment (MCI) and early-stage Alzheimer's disease (AD).

The dataset comprised 244 participants, with demographic details provided in Table 1. Voxel-based morphometry (VBM) techniques were employed to extract gray matter volume for regions of interest (ROIs). Additionally, cortical thickness values were obtained for the same ROIs. These two imaging quantitative traits (QTs) were denoted as VBM and FreeSurfer for clarity. To address potential confounding variables such as baseline age, gender, education, and handedness, the neuroimaging phenotypes were further adjusted using regression weights derived from a cohort of healthy controls (HCs). A total of 465 ROIs were defined using the MarsBaR-based Automated Anatomical Labeling (AAL) templates.

Proteomic analyte samples were assayed using the Rules Based Medicine, Inc. (RBM) proteomic panel, followed by quality control (QC) procedures. A total of 146 proteomic markers were obtained. Cognitive scores from cognitive assessments (ADAS, MMSE, FLU and so forth) were collected for the participants. Additionally, we selected 10,000 SNPs from the ADNI database and employed the additive coding paradigm, considering the count of minor alleles for the SNPs. These data processing techniques have the potential to enhance our understanding of AD and can serve as a foundation for further targeted investigations.

More details about comparison methods. (i) We search for the most related methods while most works focus on univariate or regression-based brain imaging genetics methods (TWAS, GWAS, MR, etc.) and could unravel genotype-phenotype correlations, but ignore the causal effects for diagnostic outcomes, hampering or even misleading the biological interpretation, which were inappropriate for comparison. **(ii)** Other methods, especially deep models including multiplex GNNs, CNNs, etc., are unable to identify relevant variables in the input space, let alone the causal variables hidden there. Generally, they investigate intermediate representations (layers) rather than the input variables. This post-hoc explanation cannot provide direct interpretations of the underlying

causal mechanisms of diseases. Therefore, they are unsuitable for comparison in healthcare studies. **(iii)** We compared Ca-GPCA with most related state-of-the-art models, including SMCCA, AdaSMCCA, and RelPMDCCA. These methods were the most representative of the computational imaging genetics methods and could be reduced to various specific genotype-phenotype analytical methods.

Method	SMCCA ↓ (↑)	AdaSMCCA ↓ (↑)	RelPMDCCA ↓ (↑)	Ca-GPCA ↓ (↑)
Baselines	4871 (0.17)	5018 (0.19)	6102 (0.20)	5102 (0.33)
Ours-*DC*	**52 (0.18)**	**56 (0.19)**	**61 (0.20)**	**57 (0.34)**

Table 2: Runtime (CCC). DC: **D**ivide-and-**C**onquer strategy.

Efficiency of optimization algorithm. As shown in Tab. 2 (Runtime, sec), the divide-and-conquer strategy significantly reduces time consumption by a hundredfold and holds equal performances. Specifically, Ca-GPCA reduces the computational complexity from $\mathrm{O}(np^2)$ to $\mathrm{O}(np_k^2K)$ ($p_k \ll p$, $K \ll p$)

D. Follow-up analyses for biomarker discovery

D.1. ***Follow-up analyses: Gene-set analyses***

To validate the significance of the identified SNPs, we performed a one-way analysis of covariance (ANOVA) to assess the impact of the selected SNPs on the diagnostic phenotype. As anticipated, all p-values reached statistical significance (p<0.05). To further validate the identified genetic loci with respect to biological effect at gene level, we performed gene set analyses (GSEA) using the MAGMA software. This involved a joint-SNP gene-based analysis employing a multiple linear principal components regression model. Principal components were initially derived by projecting the multivariate LD matrix of SNPs within each gene to capture genetic variations. Subsequently, Fisher's test was employed to calculate p-values, providing a measure of the association strength between a gene and the diagnostic phenotype.

Remarkably, the gene-based analysis revealed that genes including $APOE$, $APOC1$, $TOMM40$ and $PVRL2$ exhibited the highest statistical significance, indicating their substantial contributions to the diagnostic phenotype. This indicated Ca-GPCA could detect meaningful SNPs from a vast array of genetic markers. In summary, by introducing the causality module, Ca-GPCA could significantly outperform the start-of-the-art methods in identifying trustworthy causal biomarkers.

D.2. ***Follow-up analyses: Functional mapping***

To validate causal biomarker identification and facilitate interpretation, the Functional Mapping and Annotation (FUMA) platform was employed for functional mapping, prioritization, annotation and interpretation of GWAS results. Using the 1000 Genomes phase 3 reference dataset, the independent significant SNPs were identified from meta-analysis summary statistics though p-value ($p < 5 \times 10^{-8}$ *) and independence within a 1-Mb window. Subsequently, the

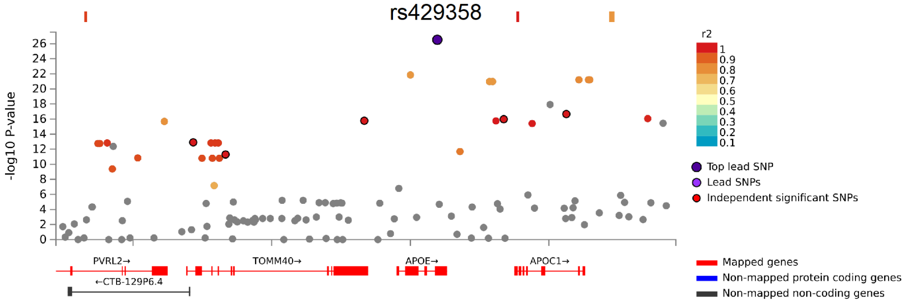

Figure 1: Summary of findings regarding the top selected SNPs, and rs429358 was detected as the top lead SNP.

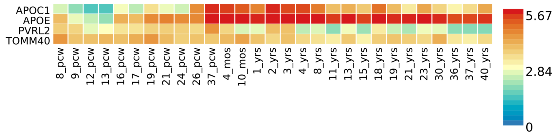

(a)

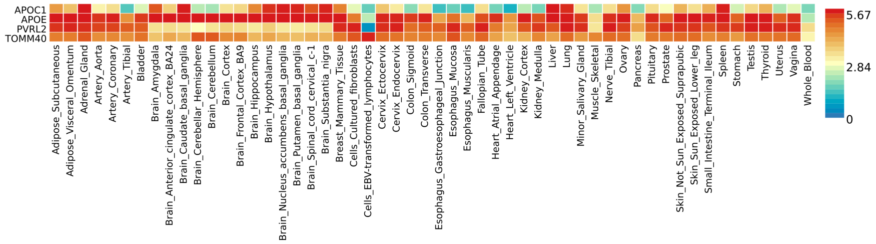

(b)

Figure 2: Heat maps illustrating the normalized gene expression values, obtained through zero mean normalization of log2-transformed expression, are presented for the prioritized genes associated with the top ten SNPs. The lower panel represents data from GTEx v8 RNAseq, while the upper panel showcases BrainSpan data.

lead SNPs were identified from these independently significant SNPs, confirming their association with target disease. Surprisingly, we observed that several genetic loci exhibited the highest level of statistical significance *i.e.* $APOE$, $APOC1$, $TOMM40$. These findings provided strong support for our Ca-GPCA results. Of note, as depicted in Figure 1, rs429358 (situated in $APOE$ on Chromosome 19) was identified as the top lead SNP, which exhibited the highest level of significance as a locus linked to AD. This finding strongly corroborated the results obtained from our Ca-GPCA, because rs429358 also exhibited the highest weight in Ca-GPCA, which reinforced the credibility and efficacy of our Ca-GPCA in identifying trustworthy biomarkers.

D.3. ***Follow-up analyses: Gene expression analyses***

To furthur assess the biological implications of the identified loci at gene expression level, we utilized the GENE2FUNC for gene expression analyses. GENE2FUNC enabled us to explore the gene expression patterns associated with the top ten SNPs. These SNPs were annotated to their corresponding genes, which served as input for our analysis. Our investigation incorporated data from the GTEx database (version 8), encompassing 54 tissue types, as well as BrainSpan RNA sequencing data covering 29 developmental stages. Heat maps were generated to visualize gene expressions, with each map representing the average normalized expression value for its respective label.

Figure 2 illustrated the mRNA expression profiles of prioritized genes associated with the top ten SNPs across all chromosomes in 54 tissue types of developing and adult human brains. The upper panel presents a heat map of gene expression based on GTEx version 8 RNA sequencing data, revealing the expression levels of genes such as $APOE$, $APOC1$, $TOMM40$, and $PVRL2$ in various brain tissues. These genes display distinct expression patterns throughout different stages of life, as observed in the BrainSpan data. Notably, $APOE$ consistently exhibited high expression levels across all lifespan stages. Conversely, APOC1 demonstrated higher expression levels during late prenatal and postnatal stages, while $PVRL2$ and $TOMM40$ shown increased expression levels during early life stages compared to later stages, which could further contribute to neurodegenerative diseases. All these findings highlighted the efficacy of our Ca-GPCA in identifying credible genetic variations associated with diverse human brain tissues throughout the lifespan.

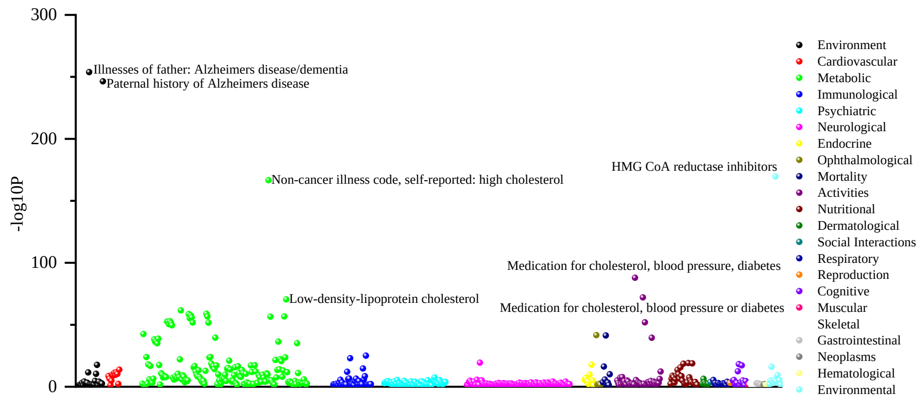

Figure 3: The PheWAS analysis yielded results for the top lead SNP.

D.4. ***Follow-up analyses: Phenome-wide association studies***

To validate the phenotypes associated with SNPs identified through Ca-GPCA, we conducted phenotype-wide association analysis (pheWAS) utilizing

Domain	Trait	p-value	N
Environment	Illnesses of father: Alzheimer's disease/dementia	1.49×10^{-254} *	355137
Environment	Paternal history of Alzheimer's disease	4.07×10^{-247} *	269279
Environmental	HMG CoA reductase inhibitors	2.60×10^{-170} *	290385
Metabolic	Non-cancer illness code, self-reported: high cholesterol	2.23×10^{-167} *	289307
Activities	Medication for cholesterol, blood pressure,diabetes	1.19×10^{-88} *	207533
Activities	Medication for cholesterol, blood pressure or diabetes: Cholesterol lowering medication	9.03×10^{-73} *	176050
Metabolic	Low-density-lipoprotein cholesterol	3.58×10^{-71} *	72866
Metabolic	Total cholesterol in LDL	2.64×10^{-62} *	21559
Metabolic	Total cholesterol in small LDL	1.24×10^{-59} *	21556
Metabolic	Total cholesterol in medium LDL	2.46×10^{-59} *	21559

Table 3: The top ten phenotypic traits were identified through PheWAS analysis.

publicly available data from GWAS Atlas32 (https://atlas.ctglab.nl). This comprehensive analysis incorporated a large dataset consisting of 4,756 GWAS, including the queried SNP or gene may not have been specifically studied. The analysis employed a Bonferroni-corrected significance threshold ($p < 1.05\times10^{-5}$) to ensure stringent statistical evaluation.

A comprehensive pheWAS analysis was conducted on the top lead SNP rs429358 to explore its potential associations across a broad spectrum of phenotypes encompassing 28 domains. The results displayed in Table 3 revealed a significant linkage between the rs429358 locus and neurological phenotypes. Specifically, associations between rs429358 and neurological traits such as Alzheimer's disease/dementia in paternal history, blood pressure, and diabetes were found to be statistically significant. Figure 3 also provided a comprehensive depiction of phenome-wide association studies for the top lead SNPs. In addition, extensive analyses shown that all of the top ten SNPs identified by Ca-GPCA were associated with neurodegenerative diseases. The combined evidence from the phenome-wide association studies underscored the successful identification of the genetic underpinnings of neurological phenotypes through our Ca-GPCA approach.

E. Phenotype Feature Explanation

Our framework was further validated through extensive experiments involving additional phenotypes. These included neuroimaging-derived phenotype markers such as LHippVol and LEntCtx, plasma-derived proteomic markers like ApoE and BNP, cerebrospinal fluid (CSF)-derived proteomic markers such as FGF-4 and VEGF, as well as cognitive markers including ADAS and MMSE. The results confirmed the associations of these markers with AD, providing further evidence of the effectiveness of our approach.

Identification and interpretation of neuroimaging-derived phenotype markers (FreeSurfer): The feature selection outcomes, illustrated in Figure 4c, presented the neuroimaging-derived phenotypes (FreeSurfer) identified through our Ca-GPCA methodology. Remarkably, Ca-GPCA assigned comparatively higher weights to a specific subset of FreeSurfer markers,*i.e.* LHippVol, LEntCtx, RMidTemporal, which have previously been reported as AD-risk makers. This consistent identification of AD-related neuroimaging-derived

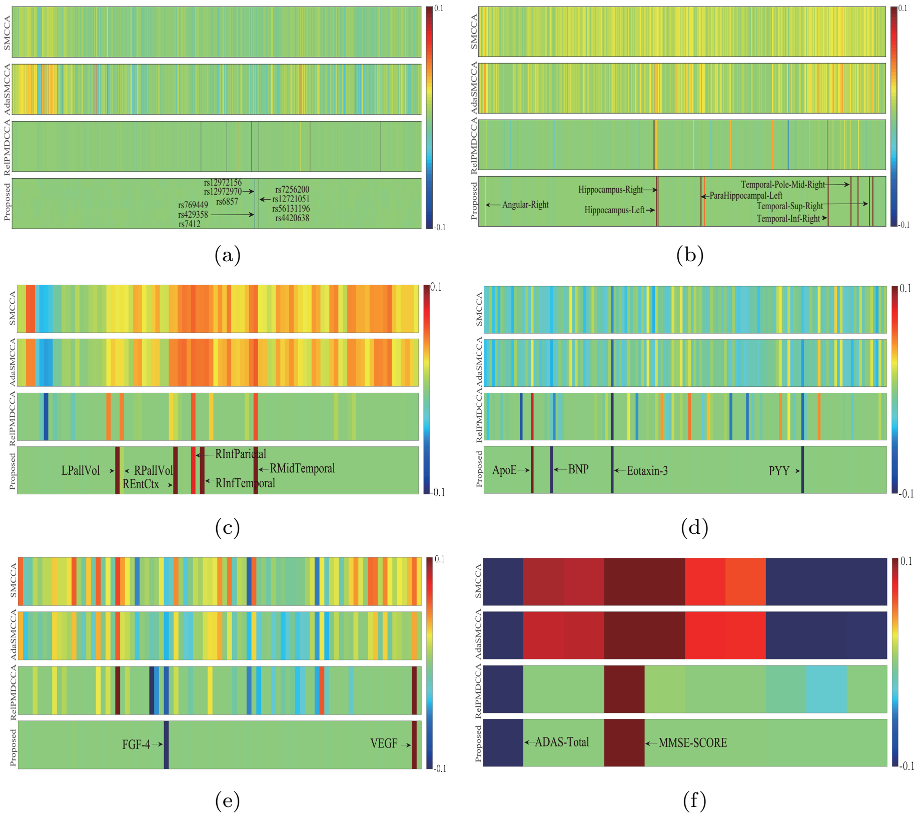

Figure 4: Biomarker discovery for SNPs and important endophenotypes. (a) SNPs. (b) Neuroimaging-derived phenotypes (VBM). (c) Neuroimaging-derived phenotypes (FreeSurfer). (d) Plasma-derived proteomic markers. (e) CSF-derived proteomic markers. (f) Cognitive score. Each row is a SCCA method: (1) SMCCA; (2) Adaptive SMCCA; (3) RelPMDCCA; (4) Proposed.

biomarkers highlighted the efficacy of Ca-GPCA in accurately and trustworthy identifying diease-related neuroimaging markers.

Identification and interpretation of plasma-derived proteomic markers: The canonical weights, depicted in Figure 4d, elucidated the importance of plasma-derived proteomic markers. By employing the causal variable decorrelation module, it was worth noting that our method successfully identified several proteomic markers associated with AD, including ApoE, BNP, Eotaxim-3, PYY and so forth. In contrast, the comparison methods also identified some AD-related proteomic markers, but they reported numerous irrelevant signals, thereby hindering the interpretation.

Identification and interpretation of CSF-derived proteomic markers: The heatmap presented in Figure 4e illustrated the canonical weights attributed to CSF-derived proteomic markers. Notably, our Ca-GPCA successfully identified AD-related CSF-derived proteomic markers, including FGF-4, VEGF and

so forth. In contrast, benchmark methods yielded an overwhelming number of markers, posing challenges for interpretation. By integrating the results from both plasma- and CSF-derived datasets, we can confidently affirm that Ca-GPCA outperformed the benchmark methods, underscoring its superior ability to identify trustworthy markers of proteomic expression.

Identification and interpretation of cognitive assessments: The canonical weights in Figure 4f emphasized the importance of cognitive markers. Interestingly, Ca-GPCA effectively identified meaningful AD-related cognitive markers, including ADAS, MMSE, which have been previously validated as important features for judging AD or not, while comparison methods identified a substantial number of markers that have not been previously reported. Overall, these findings collectively demonstrated the ability of Ca-GPCA to discern trustworthy phenotypes with significant biological implications, which could be a promising and powerful tool for multiomics brain imaging genetic studies and trustworthy causal biomarker discovery.
